# Acute lymphoblastic leukemia with e1a3 BCR/ABL fusion protein. A report of two cases

**DOI:** 10.1186/s40164-016-0049-y

**Published:** 2016-07-29

**Authors:** Bernardo López-Andrade, Francesca Sartori, Antonio Gutiérrez, Lucia García, Vanesa Cunill, María Antonia Durán, Antonia Sampol, Marta Bernués, Julio Iglesias, Rafael Ramos, Josep Lladó, María Sánchez, Juan Carlos Amat, Jordi Martínez-Serra

**Affiliations:** 1 Department of Hematology, University Hospital Son Espases, Palma de Mallorca, Spain; 2 Department of Genetics, University Hospital Son Espases, Palma de Mallorca, Spain; 3 Department of Immunology, University Hospital Son Espases, Palma de Mallorca, Spain; 4 Department of Pathology, University Hospital Son Espases, Palma de Mallorca, Spain; 5 Instituto de Investigación Sanitaria de Palma (IdISPa), Palma de Mallorca, Spain

## Abstract

B Acute Lymphoblastic leukemia (B-ALL) with Philadelphia chromosome (Ph′) is a neoplasm of lymphoblast committed to the B cell lineage. The clinical presentation of B-ALL Ph′+ is similar to B-ALL, but is more common in adults than in children. The e1a3 rare variant is produced by the fusion of BCR exon 1 to ABL exon 3. The presence of this translocation has been associated with good disease outcome for chronic myeloid leukemia in a very small series of only 5 cases; there is no such evidence for B-ALL. We report two new cases of B-ALL Ph+ with the rare e1a3 fusion transcript. The e1a3 and e1a2 (p190) transcripts have been reported to have a similar molecular weight and probably a similar clinical profile, thus in these cases the presence of e1a3 was associated with extramedullary infiltration and disease acceleration.

## Background

B Acute Lymphoblastic leukemia (B-ALL) with Philadelphia chromosome (Ph′) is a neoplasm of lymphoblast committed to the B-cell lineage. The clinical presentation of B-ALL Ph′+ is similar to B-ALL, but is more common in adults than in children and it has a worse prognosis among patients with B-ALL. The tyrosine kinase BCR-ABL fusion protein is the product of the Philadelphia chromosome, which results from the reciprocal translocation t(9;22)(q34;q11) that juxtaposes the *c*-*abl* oncogene 1 on chromosome 9 with the *bcr* gene on chromosome 22 generating the BCRABL1 oncogene [1–4]. Depending on the location of the breakpoint in BCR, several types of BCR-ABL fusion proteins may appear [5]. To date, three main breakpoint cluster regions in the BCR gene have been reported: the *M*-*bcr* region located between exons 12 and 16, the *m*-*bcr* located between exons e2′ and e2 and the *u*-*bcr* located in exon 19. The three more common variants of BCR-ABL described are p210 [more frequent in chronic myeloid leukemia (CML)], p190 (more frequent in B-ALL) and p230 (more frequent in Chronic Neutrophilic Leukemia (CNL) [6–8]. The type of rearrangement in CML is related to the patient clinical course. Other rare forms such as e1a3, b2a3 and e6a2 of BCR-ABL have been described. The e1a3 variant is produced by the fusion of BCR exon 1 to ABL exon 3. The presence of this translocation has been associated with good disease outcome for CML in a very small series of only 5 cases [9, 10]; there is no such evidence for ALL. Moreover, we have reported the first e1a3 case that progressed from CML to a lymphoid blast crisis [1]. Thus, the molecular prognosis of these rare variant forms is still unclear. We report two new cases of B-ALL Ph+ with the rare e1a3 fusion transcript.

## Case 1 presentation

**Fig. 1:**
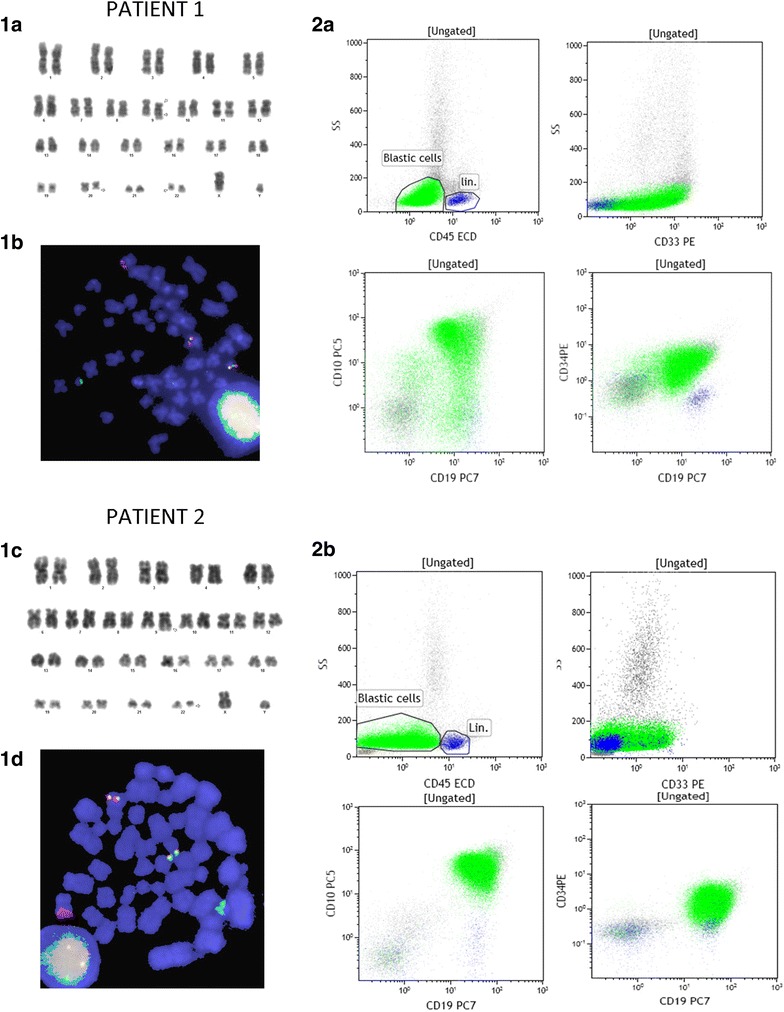
Cytogenetic, FISH and Immunophenotype results **1a** G-banded karyotype from the sideline 46,XY,del(9)(p22)t(9;22)(q34;q11),del(20)(q13). **1b** Metaphase FISH image showing BCR-ABL1 fusion gene at minor breakpoint BCR. *Green* BCR, *red* ABL1. **1c** G-banded karyotype showing t(9;22)(q34;q11) as a sole chromosome alteration. **1d** Metaphase FISH image showing BCR-ABL1 fusion gene at minor breakpoint BCR. LSI BCR/ABL1 *dual color* translocation probe (Abbott) was used. Green: BCR, Red: ABL1. **2a** Immunophenotype of ALL with atypical e1a3 translocation. Presence of 90 % of blasts with lymphoblastic phenotype B: CD19+, CD10+, CD34+, DR+, CD20+, CD22−, CD33+, cytoplasmic IgM negative and positive alpha CD79. **2b** Immunophenotype of ALL with atypical e1a3 translocation. Presence of 80 % of blasts with lymphoblastic phenotype B: CD19+, CD10+, CD34+, DR+, CD20+, CD22−, cytoplasmic IgM negative, positive alpha CD79 and dim expression of CD33

**Fig. 2:**
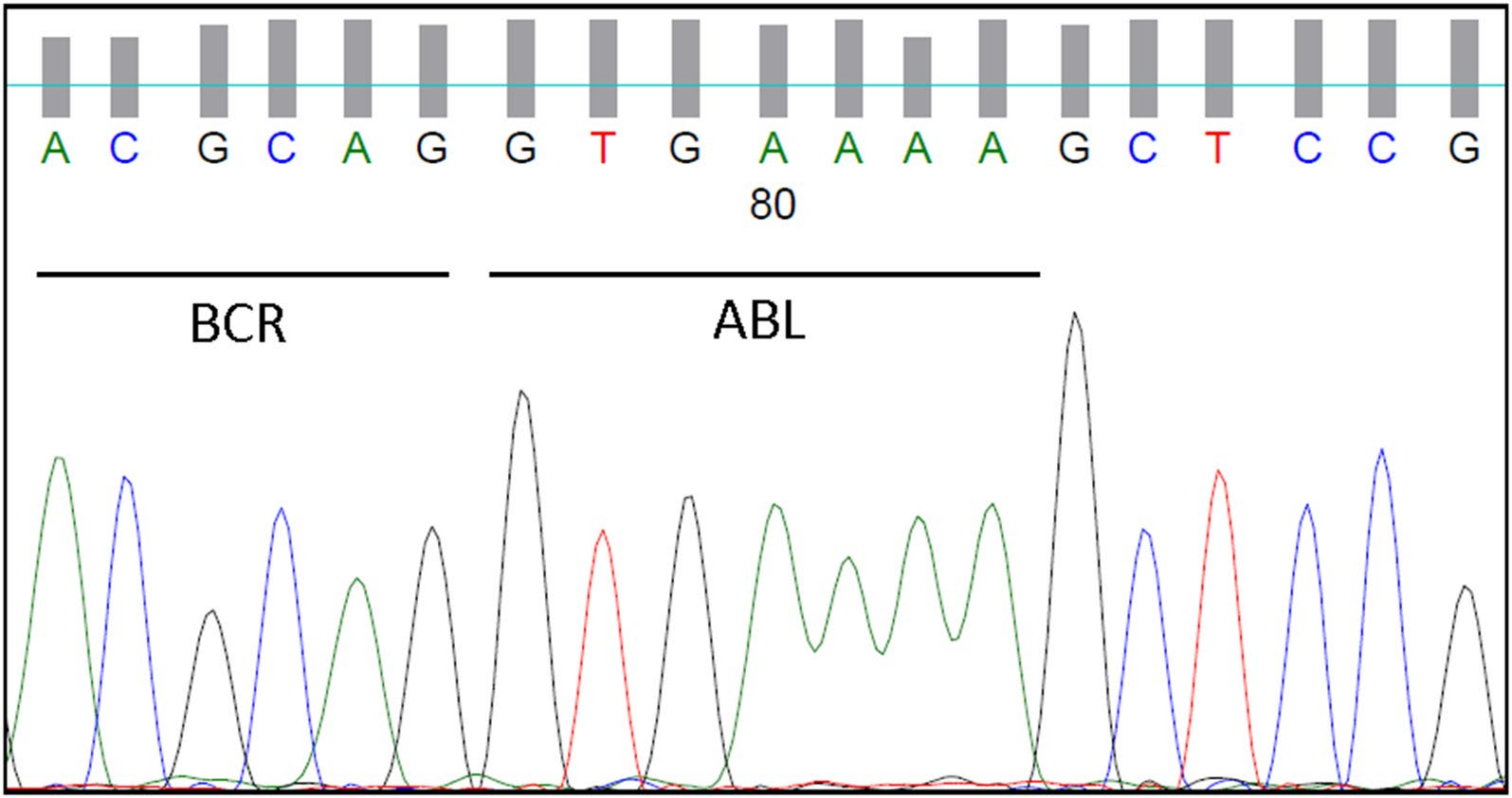
DNA sequencing of BCR-ABL e1a3 atypical amplification band purified from both patients

The first patient is a 43-year-old male, who consulted our center for night sweats and weight loss in the last 3 weeks. He had no previous history or records in our center. The physical examination was normal except for a slight splenomegaly and the blood test was compatible with hyperleukocytosis, anemia and thrombocytopenia, (leukocytes 136.00 · 10^9^/L, hemoglobin 11.70 g/dl, platelet count 39.70 · 10^9^/L) and 93 % of small-medium sized lymphoblast in the Grunwald-Giemsa blood smear. The bone marrow aspirate examination had an hypercellular infiltration of 90 % which was confirmed by immunophenotype (CD19+, CD10+, CD34+, TdT+, DR+, CD79 alfa+, CD20−, cytoplasm IgM negative). 80 % of the blast cells also expressed CD33+. A fresh sample from the bone marrow aspirate was collected for molecular and cytogenetic analysis. The assay revealed the presence of the following karyotype:46,XY,t(9;22)(q34;q11) [2] /46,XY,del(9)(p22)t(9;22)(q34;q11) [4] /46,XY,del(9)(p22)t(9;22)(q34;q11),del(20)(q13) [5] /46,XY [9]. FISH analysis confirmed the presence of BCR-ABL1 fusion gene (Fig. 1). The patient’s RNA was isolated from the peripheral blood and subjected to a two round multiplex reverse transcription and polymerase chain reaction (RT-PCR). In order to avoid RNA quality and/or handling errors, we included an internal positive control in where a 690-bp segment of the ubiquitously expressed transcription factor E2A mRNA was amplified. The primers and PCR conditions used in the first and second round of the nested PCR reaction are described by Pallisgaard et al. [11]. We identified an atypical amplification band of approximately 100 bp, much smaller than the p190 (e1a2) fragment. In order to confirm the presence of a BCR-ABL transcript this band was extracted from the agarose gel, purified and then analyzed by DNA sequencing. cDNA sequence revealed the presence of the e1a3 variant (Fig. 2).

The patient started induction treatment with ALL-type polychemotherapy and Imatinib, achieving complete response by morphology and immunophenotype. However, the BCR/ABL was still detected by RT-PCR at the end of the induction phase. He is ongoing consolidation with allogenic stem cell transplantation (SCT) from an HLA-identical sibling donor, 7 months after diagnosis.

## Case 2 presentation

The second patient is a 65-year-old male who consulted for pain in the right leg and a history of recurrent periodontal infections in the last month. An eco-doppler confirmed a thrombophlebitis and the blood test found hyperleukocytosis and anemia (leukocytes 223.00 · 10^9^/L, Hemoglobin 11.60 g/dl, platelet count 118 · 10^9^/L) and the presence of 80 % lymphoblasts in the Grunwald-Giemsa blood smear. The bone marrow aspirate found an infiltration of 25 % lymphoblast which was confirmed by immunophenotype (CD19+, CD34+, DR+, CD10+, CD22+, low intensity CD20−, TdT+, cytoplasmatic IgM negative, CD33+). The karyotype was 46,XY,t(9;22)(q34;q11) [20]. FISH analysis confirmed the presence of BCR-ABL1 fusion gene (Fig. 1). The patient’s RNA was isolated from the peripheral blood. We identified an atypical e1a3 variant after a two round PCR amplification by DNA sequencing (Fig. 2).

The patient started induction treatment and Imatinib. The bone marrow aspirate on day 34 confirmed progressive disease with 30 % blast and persistence of BCR/ABL. After a second line of intensive ALL-type induction therapy the patient finally died due to toxicity (gastrointestinal bleeding). No SCT had been planned due to age and unfit status. It is noteworthy that the blood counts in a regular check-up 4 months before the diagnosis had a slight leukocytosis that was not studied.

## Discussion

Overall only a few cases with the e1a3 BCRABL fusion proteins have been reported. This rare variant has mostly been described with an indolent clinical course in the setting of CML and its role in B-ALL is still unclear. We report two new cases of e1a3 Ph+ ALL patients in where we highlight the finding of the myeloid CD33+ marker expression by immunophenotype (biphenotypic and bilineal blast population were discarded by EGIL classification). This means that both cases could be defined as acute lymphoblastic leukemia with aberrant myeloid markers (ALL My+). We previously speculated that B-ALL e1a3 cases could represent CML in lymphoid blast crisis that underwent an under diagnosed chronic phase. In one of our cases we do not have any blood counts prior to the B-ALL diagnosis. However the other presented a non-evaluated leukocytosis in the blood count available before diagnosis.

A review of the bibliography of reported cases of B-ALL with the e1a3 fusion protein, using the terms “B-Acute lymphoblastic leukemia” and “e1a3” found a total of 18 cases plus the 2 being reported in this manuscript. The rare e1a3 translocation was also previously reported in the literature in 6 patients with CML in chronic phase. Furthermore, we described for the first time a patient with CML diagnosis that after 5 months of Imatinib treatment evolved to lymphoid blast crisis [1]. Table 1 shows all ALL cases with the e1a3 fusion protein previously reported as well as our 2 additional patients. The information provided by these reports shows that these cases were aggressive with 44 % of cases being refractory or relapsing after therapy and 55 % having already died at last follow-up.

**Table 1:** Clinical characteristics, therapy and outcome of ALL cases reported with e1a3 fusion protein compared with those presented in this manuscript

#	Age	Leukocite count at Dx	BM infiltration	TKI	SCT	Status	Response	References
1	65 years	223 × 10^3^	25 %	Imatinib	–	Dead	Refractory	Current
2	43 years	133 × 10^3^	90 %	Imatinib	AlloSCT (ongoing)	Alive	CR	Current
3	76 years	7000	85 %	Dasatinib	–	Alive	CR	Sonu [12]
4	Unk	5.530 × 10^9^/L	90 %	Imatinib	Allo SCT	Alive	CR	Shin 13]
5	Unk	24.36 × 10^9^/L	80 %	Imatinib	–	Dead	Not achieved	Shin [13]
6	80 years		94 % at blast crisis	Imatinib	–	Dead	CML transformed to B-ALL	Martinez-Serra [1]
7	62 years	NA	NA	Imatinib	–	Dead	relapse	Langabeer [14]
8	25 years	NA	NA	–	2d AlloSCT	Dead	Relapse/VOD	Fujisawa [15]
9	62 years	10	NA	–	Autologous	Dead	relapse	Burmeister [16]
10	64 years	1.8	NA	–	–	Dead	relapse	Burmeister [16]
11	31 years	31	NA	Imatinib	Allo SCT	Alive	CCR	Burmeister [16]
12	61years	14	NA	Imatinib	–	Alive	CCR	Burmeister [16]
13	48 years	62	NA	–	–	Dead	Relapse	Burmeister [16]
14	37 years	70	NA	Imatinib	AlloSCT	Alive	CCR	Burmeister [16]
15	45 years	5	NA	Imatinib	AlloSCT	Alive	CCR	Burmeister [16]
16	29 years	40	NA	Imatinib	AlloSCT	Alive	CCR	Burmeister [16]
17	NA	NA	NA	NA	NA	NA	NA	Wilson [17]
18	1 year	NA	NA	NA	NA	Dead	NA	Iwata [18]
19	39 years	NA	NA	NA	NA	Dead	NA	Soakerman [19]
20	NA	NA	NA	NA	NA	NA	NA	Chen [20]

*B-ALL* B-lymphoblastic leukemia/lymphoma, *CML* chronic myeloid leukemia, *Allo SCT* allogeneic hematopoietic stem cell transplantation, *CCR* clinical complete remission, *TKI* tyrosine kinase inhibitor, *NA* not available

The review of our cases and the other previously reported shows an aggressive disease similar to other Ph+ ALL. More data is needed in order to clearly define the real prognosis of B-ALL cases with e1a3 translocation and state if these patients could benefit from TKI or other approaches such as Allo SCT at consolidation. The e1a3 and e1a2 (p190) transcripts have been reported to have a similar molecular weight and probably a similar clinical profile, meaning that e1a3 could be associated with the presence of extramedullary infiltration and disease acceleration, with both patients (one achieving CR and one with PD) having persistence of e1a3 at the molecular level after induction treatment. Moreover, it is important that our reference laboratory is able to detect the rare e1a3 BCR-ABL avoiding false e1a3 negatives, in both ALL and CML.

## Conclusion

We report 2 new cases of B-ALL with the rare e1a3 BCR-ABL fusion protein. At the moment the particular prognostic significance of e1a3 remains undetermined, but considering our cases and the other previously described, it seems that a clinical management with protocols of Ph+ ALL should be offered to eligible patients. The study of the clinical significance of these variants at diagnosis and follow up, is important to define if these patients have a higher risk of relapse or progressive disease in comparison to other BCR/ABL transcripts and could benefit from SCT.

## Abbreviations

B-ALL: B Acute Lymphoblastic Leukemia B-ALL
Ph′: Philadelphia chromosome
CML: chronic myeloid leukemia
CNL: chronic neutrophilic leukemia
RT-PCR: reverse transcription and polymerase chain reaction
FISH: fluorescence in situ hybridization
Allo SCT: allogeneic hematopoietic stem cell transplantation
TKI: tyrosine kinase inhibitor
CR: complete remission
PD: progressive disease

## References

[1] Martinez-Serra J, Del Campo R, Gutierrez A, Antich JL, Ginard M, Durán MA, et al. Chronic myeloid leukemia with an e1a3 BCR-ABL fusion protein: transformation to lymphoid blast crisis. Biomark Res. 2014;2:14. http://www.pubmedcentral.nih.gov/articlerender.fcgi?artid=4155769&tool=pmcentrez&rendertype=abstract. 10.1186/2050-7771-2-14 PMC4155769 25197554

[2] Nowell PC, Hungerford DA (1960). Chromosome studies on normal and leukemic human leukocytes. J Natl Cancer Inst.

[3] Prieto F, Egozcue J, Forteza G, Marco F. Identification of the Philadelphia (Ph-1) chromosome. Blood. 1970;35(1):23–7. http://www.ncbi.nlm.nih.gov/pubmed/5263119. 5263119

[4] Rowley JD. Molecular analysis of rearrangements in Philadelphia (Ph1) chromosome-positive leukemia. Haematol Blood Transfus. 1989;32(32):3–10. http://www.ncbi.nlm.nih.gov/pubmed/2696684. 10.1007/978-3-642-74621-5_1 2696684

[5] Deininger MWN, Goldman JM, Melo JV, Dc W, Deininger MWN, Goldman JM (2013). The molecular biology of chronic myeloid leukemia. Blood.

[6] Laurent E, Talpaz M, Kantarjian H, Kurzrock R (2001). The BCR gene and Philadelphia chromosome-positive leukemogenesis. Cancer Res.

[7] Heisterkamp N, Stam K, Groffen J, de Klein A, Grosveld G (1985). Structural organization of the bcr gene and its role in the Ph′ translocation. Nature.

[8] Pane F, Frigeri F, Sindona M, Luciano L, Ferrara F, Cimino R (1996). Neutrophilic-chronic myeloid leukemia: a distinct disease with a specific molecular marker (BCR/ABL with C3/A2 junction). Blood.

[9] Al-Ali HK, Leiblein S, Kovacs I, Hennig E, Niederwieser D, Deininger MWN (2002). CML with an e1a3 BCR-ABL fusion: rare, benign, and a potential diagnostic pitfall. Blood.

[10] Roman J, Jimenez A, Barrios M, Castillejo JA, Maldonado J, Torres A (2001). E1A3 as a unique, naturally occurring BCR-ABL transcript in an indolent case of chronic myeloid leukaemia. Br J Haematol.

[11] Pallisgaard N, Hokland P, Riishøj DC, Pedersen B, Jørgensen P. Multiplex reverse transcription-polymerase chain reaction for simultaneous screening of 29 translocations and chromosomal aberrations in acute leukemia. Blood. 1998;92(2):574–88. http://www.ncbi.nlm.nih.gov/pubmed/9657758. 9657758

[12] Sonu RJ, Jonas BA, Dwyre DM, Gregg JP, Rashidi HH. Optimal molecular methods in detecting p190 BCR-ABL fusion variants in hematologic malignancies: a case report and review of the literature. Case Rep Hematol. 2015;2015:1–6. http://www.hindawi.com/journals/crihem/2015/458052/. 10.1155/2015/458052 PMC4407518 25949834

[13] Shin SY, Cho JH, Kim HJ, Jang JH, Lee ST, Kim SH (2015). Two cases of acute lymphoblastic leukemia with an e1a3 BCR-ABL1 fusion transcript. Ann Lab Med.

[14] Langabeer SE, Haslam K, Kelly J, Leahy M, Vandenberghe E (2011). Acute lymphoblastic leukaemia with an e1a3 BCR-ABL1 fusion. Acta Haematol.

[15] Fujisawa S, Nakamura S, Naito K, Kobayashi M, Ohnishi K (2008). A variant transcript, e1a3, of the minor BCR-ABL fusion gene in acute lymphoblastic leukemia: case report and review of the literature. Int J Hematol.

[16] Burmeister T, Schwartz S, Taubald A, Jost E, Lipp T, Schneller F (2007). Atypical BCR-ABL mRNA transcripts in adult acute lymphoblastic leukemia. Haematologica.

[17] Wilson GA, Vandenberghe EA, Pollitt RC, Rees DC, Goodeve AC, Peake IR (2000). Are aberrant BCR-ABL transcripts more common than previously thought?. Br J Haematol.

[18] Iwata S, Mizutani S, Nakazawa S, Yata J (1994). Heterogeneity of the breakpoint in the ABL gene in cases with BCR/ABL transcript lacking ABL exon a2. Leukemia.

[19] Soekarman D, van Denderen J, Hoefsloot L, Moret M, Meeuwsen T, van Baal J, et al. A novel variant of the bcr-abl fusion product in Philadelphia chromosome-positive acute lymphoblastic leukemia. Leukemia. 1990;4(6):397–403. http://www.ncbi.nlm.nih.gov/pubmed/2193202. 2193202

[20] Chen Y, Wang HW, Chen XH, Xu ZF, Qin YH, Ren FG, et al. Adult acute lymphoblastic leukemia with atypical BCR-ABL transcript e1a3: a case report and literature review. Zhonghua Xue Ye Xue Za Zhi. 2013;34(11):965–6. http://www.ncbi.nlm.nih.gov/pubmed/24294854. 10.3760/cma.j.issn.0253-2727.2013.11.012 24294854

